# (2*E*)-3-(2-Bromo­phen­yl)-1-(4,4′′-difluoro-5′-meth­oxy-1,1′:3′,1′′-terphenyl-4′-yl)prop-2-en-1-one

**DOI:** 10.1107/S1600536812013852

**Published:** 2012-04-06

**Authors:** Hoong-Kun Fun, Tze Shyang Chia, S. Samshuddin, B. Narayana, B. K. Sarojini

**Affiliations:** a X-ray Crystallography Unit, School of Physics, Universiti Sains Malaysia, 11800 USM, Penang, Malaysia; b Department of Studies in Chemistry, Mangalore University, Mangalagangotri 574 199, India; c Department of Chemistry, P. A. College of Engineering, Nadupadavu, Mangalore 574 153, India

## Abstract

In the title compound, C_28_H_19_BrF_2_O_2_, the central benzene ring makes dihedral angles of 62.51 (18), 46.23 (18) and 48.19 (18)° with the bromo-substituted benzene ring and two terminal fluoro-substituted benzene rings, respectively. In the crystal, mol­ecules are linked by C—H⋯F hydrogen bonds into infinite chains along [110]. Weak C—H⋯π and π–π inter­actions [centroid–centroid distance = 3.683 (2) Å] also occur and short inter­molecular F⋯F contacts [2.833 (4) Å] are observed.

## Related literature

For related structures and background to terphenyl chalcones, see: Fun *et al.* (2011*a* ▶,*b* ▶, 2012 ▶). For reference bond lengths, see: Allen *et al.* (1987 ▶). For the stability of the temperature controller used in the data collection, see: Cosier & Glazer (1986 ▶).
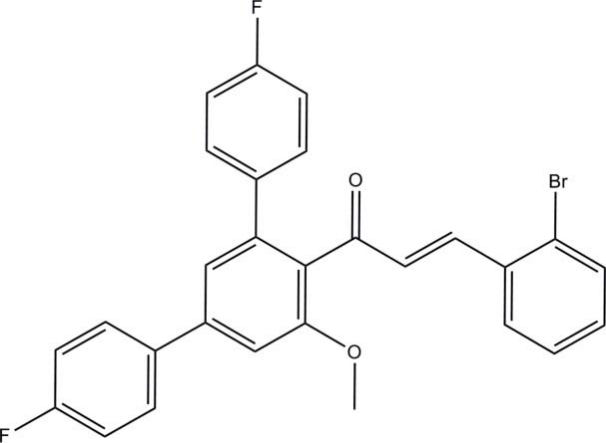


## Experimental

### 

#### Crystal data

C_28_H_19_BrF_2_O_2_
*M* *_r_* = 505.34
Monoclinic, 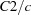

*a* = 22.4861 (6) Å
*b* = 6.9006 (2) Å
*c* = 28.6933 (8) Å
β = 101.286 (2)°
*V* = 4366.2 (2) Å^3^
*Z* = 8
Mo *K*α radiation
μ = 1.92 mm^−1^
*T* = 100 K
0.37 × 0.12 × 0.08 mm

#### Data collection

Bruker SMART APEXII CCD diffractometer
Absorption correction: multi-scan (*SADABS*; Bruker, 2009 ▶) *T*_min_ = 0.533, *T*_max_ = 0.863
24190 measured reflections
6414 independent reflections
4483 reflections with *I* > 2σ(*I*)
*R*_int_ = 0.076

#### Refinement

*R*[*F* ^2^ > 2σ(*F* ^2^)] = 0.071
*wR*(*F* ^2^) = 0.134
*S* = 1.11
6414 reflections
299 parameters
H-atom parameters constrained
Δρ_max_ = 0.72 e Å^−3^
Δρ_min_ = −1.09 e Å^−3^

### 

Data collection: *APEX2* (Bruker, 2009 ▶); cell refinement: *SAINT* (Bruker, 2009 ▶); data reduction: *SAINT*; program(s) used to solve structure: *SHELXTL* (Sheldrick, 2008 ▶); program(s) used to refine structure: *SHELXTL*; molecular graphics: *SHELXTL*; software used to prepare material for publication: *SHELXTL* and *PLATON* (Spek, 2009 ▶).

## Supplementary Material

Crystal structure: contains datablock(s) global, I. DOI: 10.1107/S1600536812013852/hb6704sup1.cif

Structure factors: contains datablock(s) I. DOI: 10.1107/S1600536812013852/hb6704Isup2.hkl

Supplementary material file. DOI: 10.1107/S1600536812013852/hb6704Isup3.cml

Additional supplementary materials:  crystallographic information; 3D view; checkCIF report

## Figures and Tables

**Table 1:** Hydrogen-bond geometry (Å, °) *Cg*1 and *Cg*2 are the centroids of C1—C6 and C10—C15 rings, respectively.

*D*—H⋯*A*	*D*—H	H⋯*A*	*D*⋯*A*	*D*—H⋯*A*
C28—H28*A*⋯F2^i^	0.96	2.51	3.448 (4)	166
C4—H4*A*⋯*Cg*1^ii^	0.93	2.99	3.712 (5)	136
C20—H20*A*⋯*Cg*2^iii^	0.93	2.72	3.383 (4)	129
C27—H27*A*⋯*Cg*1^iv^	0.93	2.95	3.735 (4)	143
C28—H28*B*⋯*Cg*2^v^	0.96	2.82	3.485 (4)	128

Symmetry codes: (i) 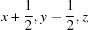; (ii) 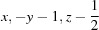; (iii) 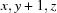; (iv) 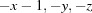; (v) 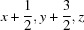.

**Fig. 1.**
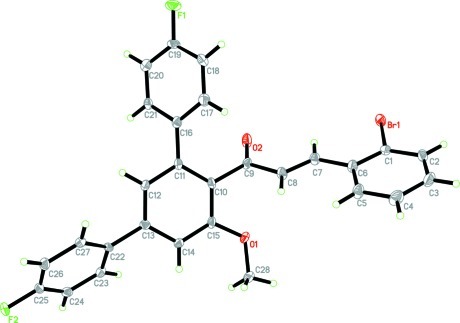
The molecular structure of the title compound with 50% probability displacement ellipsoids.

**Fig. 2.**
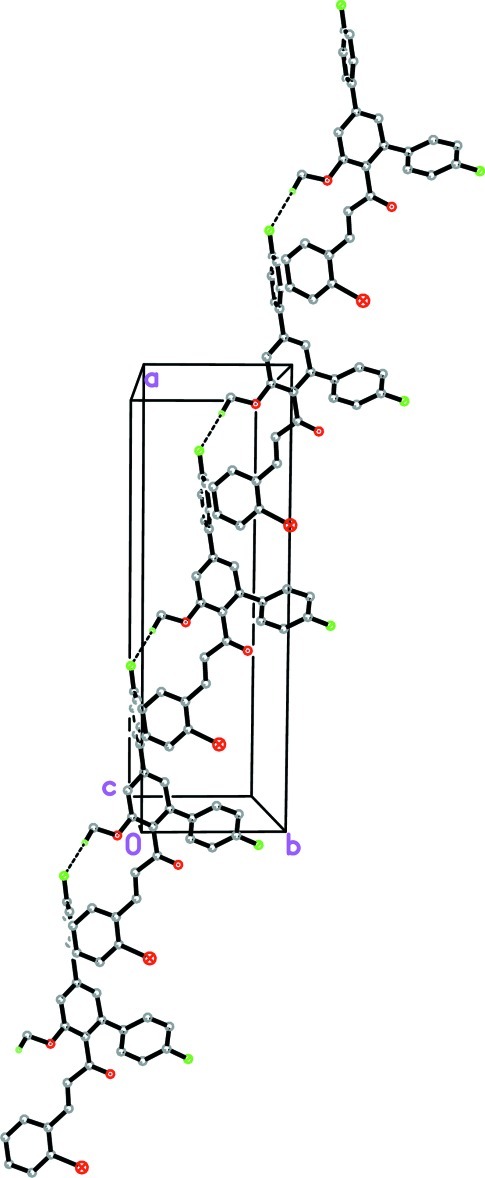
The crystal packing of the title compound. The dashed lines represent the hydrogen bonds.

## supplementary crystallographic information

### Comment

In continuation of our work on the synthesis and structures of terphenyl chalcones (Fun *et al.*, 2011*a*,*b*), the title compound (I) is now described. The starting material of the title compound was prepared from 4,4'-difluoro chalcone by several steps (Fun *et al.*, 2012).

In the title compound (Fig. 1), the central benzene ring (C10–C15) makes dihedral angles of 62.51 (18), 46.23 (18) and 48.19 (18)° with the bromo-substituted benzene ring (C1–C6) and two terminal fluoro-substituted benzene rings (C16–C21 & C22–C27), respectively. Bond lengths (Allen *et al.*, 1987) and angles are within normal ranges and are comparable to related structures (Fun *et al.*, 2011*a*,*b*, 2012).

In the crystal (Fig. 2), molecules are linked by C28—H28A···F2 hydrogen bonds into infinite chains along [110]. The crystal is further stabilized by C—H···π interactions (Table 1), involving *Cg*1 and *Cg*2 which are the centroids of C1—C6 and C10—C15 rings, respectively. π–π interaction is also observed with *Cg*4···*Cg*4 distance of 3.683 (2) Å [symmetry code: -1/2-*X*,3/2-Y,-*Z*], where *Cg*4 is the centroid of C22–C27 ring.

### Experimental

To a mixture of 1-(4,4''-difluoro-5'-methoxy-1,1':3',1''-terphenyl-4'-yl) ethanone (0.338 g, 0.001 mol) and 2-bromobenzaldehyde (0.185 g, 0.001 mol) in 30 ml e thanol, 0.5 ml of 10% sodium hydroxide solution was added and stirred at 5–10 °C for 3 h. The precipitate formed was collected by filtration and purified by recrystallization from ethanol. Colourless needles were grown from DMF solution by slow evaporation method and yield of the compound was 79%. (m.p.: 440 K).

### Refinement

All H atoms were positioned geometrically [C—H = 0.93 and 0.96 Å] and refined using a riding model with *U*_iso_(H) = 1.2 or 1.5*U*_eq_(C). A rotating group model was applied to the methyl group.

### Crystal data

**Table:**

C_28_H_19_BrF_2_O_2_	*F*(000) = 2048
*M**_r_* = 505.34	*D*_x_ = 1.538 Mg m^−^^3^
Monoclinic, *C*2/*c*	Mo *K*α radiation, λ = 0.71073 Å
Hall symbol: -C 2yc	Cell parameters from 4738 reflections
*a* = 22.4861 (6) Å	θ = 2.6–29.4°
*b* = 6.9006 (2) Å	µ = 1.92 mm^−^^1^
*c* = 28.6933 (8) Å	*T* = 100 K
β = 101.286 (2)°	Needle, colourless
*V* = 4366.2 (2) Å^3^	0.37 × 0.12 × 0.08 mm
*Z* = 8	

### Data collection

**Table:**

Bruker SMART APEXII CCD diffractometer	6414 independent reflections
Radiation source: fine-focus sealed tube	4483 reflections with *I* > 2σ(*I*)
Graphite monochromator	*R*_int_ = 0.076
φ and ω scans	θ_max_ = 30.3°, θ_min_ = 1.5°
Absorption correction: multi-scan (*SADABS*; Bruker, 2009)	*h* = −31→23
*T*_min_ = 0.533, *T*_max_ = 0.863	*k* = −9→9
24190 measured reflections	*l* = −40→39

### Refinement

**Table:**

Refinement on *F*^2^	Primary atom site location: structure-invariant direct methods
Least-squares matrix: full	Secondary atom site location: difference Fourier map
*R*[*F*^2^ > 2σ(*F*^2^)] = 0.071	Hydrogen site location: inferred from neighbouring sites
*wR*(*F*^2^) = 0.134	H-atom parameters constrained
*S* = 1.11	*w* = 1/[σ^2^(*F*_o_^2^) + (0.0321*P*)^2^ + 25.8799*P*] where *P* = (*F*_o_^2^ + 2*F*_c_^2^)/3
6414 reflections	(Δ/σ)_max_ < 0.001
299 parameters	Δρ_max_ = 0.72 e Å^−^^3^
0 restraints	Δρ_min_ = −1.09 e Å^−^^3^

### Special details

**Table:**

Experimental. The crystal was placed in the cold stream of an Oxford Cryosystems Cobra open-flow nitrogen cryostat (Cosier & Glazer, 1986) operating at 100.0 (1) K.
Geometry. All e.s.d.'s (except the e.s.d. in the dihedral angle between two l.s. planes) are estimated using the full covariance matrix. The cell e.s.d.'s are taken into account individually in the estimation of e.s.d.'s in distances, angles and torsion angles; correlations between e.s.d.'s in cell parameters are only used when they are defined by crystal symmetry. An approximate (isotropic) treatment of cell e.s.d.'s is used for estimating e.s.d.'s involving l.s. planes.
Refinement. Refinement of *F*^2^ against ALL reflections. The weighted *R*-factor *wR* and goodness of fit *S* are based on *F*^2^, conventional *R*-factors *R* are based on *F*, with *F* set to zero for negative *F*^2^. The threshold expression of *F*^2^ > σ(*F*^2^) is used only for calculating *R*-factors(gt) *etc*. and is not relevant to the choice of reflections for refinement. *R*-factors based on *F*^2^ are statistically about twice as large as those based on *F*, and *R*- factors based on ALL data will be even larger.

### Fractional atomic coordinates and isotropic or equivalent isotropic displacement parameters (Å^2^)

**Table:**

	*x*	*y*	*z*	*U*_iso_*/*U*_eq_	
F1	0.05500 (11)	1.3787 (4)	0.23294 (8)	0.0264 (6)	
F2	−0.34855 (10)	0.4660 (3)	−0.01133 (9)	0.0242 (5)	
Br1	0.327887 (17)	0.59947 (6)	0.161236 (15)	0.02040 (11)	
O1	0.04875 (11)	0.3643 (4)	0.05277 (10)	0.0186 (6)	
O2	0.11379 (12)	0.8145 (4)	0.10429 (10)	0.0227 (6)	
C1	0.29189 (17)	0.3696 (6)	0.17939 (13)	0.0164 (8)	
C2	0.32983 (18)	0.2283 (6)	0.20296 (13)	0.0205 (9)	
H2A	0.3716	0.2470	0.2094	0.025*	
C3	0.30549 (19)	0.0591 (6)	0.21700 (15)	0.0235 (9)	
H3A	0.3308	−0.0355	0.2333	0.028*	
C4	0.2435 (2)	0.0309 (6)	0.20675 (16)	0.0252 (10)	
H4A	0.2271	−0.0833	0.2160	0.030*	
C5	0.20594 (18)	0.1712 (6)	0.18288 (15)	0.0217 (9)	
H5A	0.1644	0.1484	0.1755	0.026*	
C6	0.22854 (17)	0.3485 (6)	0.16924 (14)	0.0175 (8)	
C7	0.18826 (17)	0.5015 (6)	0.14592 (14)	0.0171 (8)	
H7A	0.2064	0.6160	0.1389	0.020*	
C8	0.12818 (16)	0.4921 (6)	0.13392 (14)	0.0162 (8)	
H8A	0.1087	0.3784	0.1398	0.019*	
C9	0.09102 (16)	0.6582 (6)	0.11128 (14)	0.0152 (8)	
C10	0.02379 (15)	0.6264 (5)	0.09697 (13)	0.0134 (7)	
C11	−0.01873 (16)	0.7463 (5)	0.11261 (13)	0.0132 (7)	
C12	−0.08046 (16)	0.7099 (5)	0.09609 (13)	0.0131 (7)	
H12A	−0.1089	0.7873	0.1068	0.016*	
C13	−0.10049 (16)	0.5610 (5)	0.06407 (13)	0.0130 (7)	
C14	−0.05778 (16)	0.4412 (5)	0.04891 (13)	0.0140 (8)	
H14A	−0.0705	0.3409	0.0277	0.017*	
C15	0.00371 (16)	0.4725 (6)	0.06567 (14)	0.0149 (8)	
C16	−0.00013 (15)	0.9085 (6)	0.14622 (13)	0.0149 (7)	
C17	0.04441 (17)	0.8892 (6)	0.18743 (14)	0.0195 (8)	
H17A	0.0624	0.7691	0.1952	0.023*	
C18	0.06198 (17)	1.0457 (6)	0.21673 (14)	0.0183 (8)	
H18A	0.0915	1.0315	0.2442	0.022*	
C19	0.03536 (17)	1.2232 (6)	0.20488 (14)	0.0168 (8)	
C20	−0.01063 (17)	1.2479 (6)	0.16598 (14)	0.0170 (8)	
H20A	−0.0298	1.3671	0.1596	0.020*	
C21	−0.02756 (15)	1.0898 (6)	0.13661 (13)	0.0146 (7)	
H21A	−0.0580	1.1046	0.1098	0.018*	
C22	−0.16673 (16)	0.5323 (5)	0.04452 (13)	0.0129 (7)	
C23	−0.18668 (17)	0.5112 (6)	−0.00430 (14)	0.0167 (8)	
H23A	−0.1588	0.5115	−0.0243	0.020*	
C24	−0.24795 (17)	0.4900 (5)	−0.02318 (14)	0.0172 (8)	
H24A	−0.2616	0.4772	−0.0558	0.021*	
C25	−0.28826 (17)	0.4882 (6)	0.00758 (15)	0.0176 (8)	
C26	−0.27040 (17)	0.5068 (6)	0.05564 (15)	0.0182 (8)	
H26A	−0.2986	0.5034	0.0754	0.022*	
C27	−0.20866 (17)	0.5311 (5)	0.07448 (14)	0.0150 (8)	
H27A	−0.1955	0.5466	0.1071	0.018*	
C28	0.03253 (18)	0.1869 (6)	0.02761 (15)	0.0206 (9)	
H28A	0.0686	0.1224	0.0226	0.031*	
H28B	0.0068	0.2145	−0.0025	0.031*	
H28C	0.0113	0.1049	0.0459	0.031*	

### Atomic displacement parameters (Å^2^)

**Table:**

	*U*^11^	*U*^22^	*U*^33^	*U*^12^	*U*^13^	*U*^23^
F1	0.0268 (13)	0.0204 (13)	0.0315 (14)	−0.0099 (11)	0.0043 (10)	−0.0100 (11)
F2	0.0090 (10)	0.0241 (13)	0.0374 (14)	0.0011 (9)	−0.0004 (10)	−0.0055 (11)
Br1	0.00986 (16)	0.0217 (2)	0.0293 (2)	−0.00161 (18)	0.00283 (13)	−0.00163 (19)
O1	0.0135 (12)	0.0155 (14)	0.0275 (15)	0.0042 (11)	0.0056 (11)	−0.0053 (11)
O2	0.0125 (13)	0.0180 (15)	0.0377 (18)	0.0013 (12)	0.0054 (12)	0.0066 (13)
C1	0.0171 (18)	0.017 (2)	0.0142 (18)	0.0018 (15)	0.0005 (14)	−0.0030 (15)
C2	0.0183 (19)	0.026 (2)	0.015 (2)	0.0079 (17)	−0.0029 (15)	−0.0044 (17)
C3	0.028 (2)	0.019 (2)	0.023 (2)	0.0101 (18)	0.0023 (17)	0.0017 (17)
C4	0.028 (2)	0.016 (2)	0.033 (3)	0.0005 (18)	0.0101 (19)	0.0042 (18)
C5	0.0141 (18)	0.019 (2)	0.033 (2)	0.0015 (16)	0.0076 (17)	0.0045 (18)
C6	0.0157 (18)	0.016 (2)	0.020 (2)	0.0028 (15)	0.0029 (15)	0.0022 (15)
C7	0.0131 (17)	0.018 (2)	0.021 (2)	0.0006 (16)	0.0046 (15)	0.0023 (16)
C8	0.0104 (17)	0.018 (2)	0.022 (2)	−0.0023 (15)	0.0057 (15)	0.0029 (16)
C9	0.0111 (17)	0.0147 (19)	0.022 (2)	0.0018 (14)	0.0080 (15)	0.0003 (15)
C10	0.0082 (15)	0.0132 (19)	0.0184 (19)	−0.0001 (14)	0.0017 (13)	0.0036 (15)
C11	0.0102 (16)	0.0144 (19)	0.0146 (18)	0.0022 (14)	0.0013 (14)	0.0022 (15)
C12	0.0115 (16)	0.0111 (18)	0.0176 (19)	0.0017 (15)	0.0051 (14)	0.0014 (14)
C13	0.0132 (16)	0.0105 (18)	0.0148 (18)	−0.0021 (14)	0.0015 (14)	0.0005 (14)
C14	0.0137 (17)	0.0102 (19)	0.0181 (19)	0.0008 (14)	0.0034 (14)	−0.0004 (14)
C15	0.0097 (16)	0.0136 (18)	0.022 (2)	0.0015 (14)	0.0060 (15)	0.0028 (15)
C16	0.0113 (16)	0.0156 (18)	0.0192 (19)	−0.0009 (16)	0.0064 (14)	0.0009 (16)
C17	0.0166 (18)	0.017 (2)	0.024 (2)	0.0006 (17)	0.0023 (15)	−0.0001 (17)
C18	0.0131 (17)	0.021 (2)	0.019 (2)	−0.0023 (16)	−0.0007 (15)	0.0005 (16)
C19	0.0161 (18)	0.0149 (19)	0.021 (2)	−0.0087 (16)	0.0068 (15)	−0.0065 (16)
C20	0.0149 (18)	0.0127 (19)	0.026 (2)	−0.0039 (15)	0.0109 (16)	0.0009 (16)
C21	0.0098 (15)	0.0173 (19)	0.0176 (18)	0.0006 (16)	0.0044 (13)	0.0006 (16)
C22	0.0104 (16)	0.0082 (17)	0.020 (2)	−0.0028 (14)	0.0026 (14)	−0.0002 (14)
C23	0.0154 (18)	0.0137 (19)	0.021 (2)	0.0025 (15)	0.0041 (15)	−0.0013 (16)
C24	0.0182 (19)	0.0120 (19)	0.019 (2)	0.0019 (16)	−0.0017 (16)	−0.0011 (15)
C25	0.0112 (17)	0.0096 (18)	0.030 (2)	0.0011 (15)	−0.0007 (16)	−0.0008 (16)
C26	0.0128 (18)	0.016 (2)	0.027 (2)	−0.0007 (16)	0.0084 (16)	−0.0004 (17)
C27	0.0150 (18)	0.0113 (18)	0.018 (2)	0.0007 (15)	0.0027 (15)	−0.0013 (15)
C28	0.0177 (19)	0.017 (2)	0.028 (2)	0.0032 (16)	0.0070 (17)	−0.0049 (17)

### Geometric parameters (Å, º)

**Table:**

F1—C19	1.362 (4)	C13—C14	1.400 (5)
F2—C25	1.366 (4)	C13—C22	1.499 (5)
Br1—C1	1.899 (4)	C14—C15	1.389 (5)
O1—C15	1.366 (4)	C14—H14A	0.9300
O1—C28	1.432 (5)	C16—C21	1.398 (5)
O2—C9	1.227 (5)	C16—C17	1.398 (5)
C1—C2	1.382 (5)	C17—C18	1.378 (6)
C1—C6	1.405 (5)	C17—H17A	0.9300
C2—C3	1.383 (6)	C18—C19	1.377 (6)
C2—H2A	0.9300	C18—H18A	0.9300
C3—C4	1.380 (6)	C19—C20	1.375 (5)
C3—H3A	0.9300	C20—C21	1.386 (5)
C4—C5	1.377 (6)	C20—H20A	0.9300
C4—H4A	0.9300	C21—H21A	0.9300
C5—C6	1.409 (6)	C22—C23	1.392 (5)
C5—H5A	0.9300	C22—C27	1.395 (5)
C6—C7	1.465 (5)	C23—C24	1.386 (5)
C7—C8	1.329 (5)	C23—H23A	0.9300
C7—H7A	0.9300	C24—C25	1.384 (6)
C8—C9	1.490 (5)	C24—H24A	0.9300
C8—H8A	0.9300	C25—C26	1.365 (6)
C9—C10	1.503 (5)	C26—C27	1.398 (5)
C10—C11	1.403 (5)	C26—H26A	0.9300
C10—C15	1.407 (5)	C27—H27A	0.9300
C11—C12	1.399 (5)	C28—H28A	0.9600
C11—C16	1.483 (5)	C28—H28B	0.9600
C12—C13	1.393 (5)	C28—H28C	0.9600
C12—H12A	0.9300		
			
C15—O1—C28	118.3 (3)	O1—C15—C10	115.0 (3)
C2—C1—C6	122.1 (4)	C14—C15—C10	120.8 (3)
C2—C1—Br1	117.9 (3)	C21—C16—C17	117.9 (4)
C6—C1—Br1	120.0 (3)	C21—C16—C11	119.3 (3)
C1—C2—C3	119.8 (4)	C17—C16—C11	122.8 (4)
C1—C2—H2A	120.1	C18—C17—C16	120.9 (4)
C3—C2—H2A	120.1	C18—C17—H17A	119.6
C4—C3—C2	119.8 (4)	C16—C17—H17A	119.6
C4—C3—H3A	120.1	C19—C18—C17	119.2 (4)
C2—C3—H3A	120.1	C19—C18—H18A	120.4
C5—C4—C3	120.2 (4)	C17—C18—H18A	120.4
C5—C4—H4A	119.9	F1—C19—C20	119.2 (4)
C3—C4—H4A	119.9	F1—C19—C18	118.7 (3)
C4—C5—C6	122.0 (4)	C20—C19—C18	122.1 (4)
C4—C5—H5A	119.0	C19—C20—C21	118.0 (4)
C6—C5—H5A	119.0	C19—C20—H20A	121.0
C1—C6—C5	116.0 (3)	C21—C20—H20A	121.0
C1—C6—C7	122.1 (4)	C20—C21—C16	121.8 (3)
C5—C6—C7	121.9 (3)	C20—C21—H21A	119.1
C8—C7—C6	126.1 (4)	C16—C21—H21A	119.1
C8—C7—H7A	116.9	C23—C22—C27	119.7 (3)
C6—C7—H7A	116.9	C23—C22—C13	119.4 (3)
C7—C8—C9	122.0 (4)	C27—C22—C13	120.8 (3)
C7—C8—H8A	119.0	C24—C23—C22	120.3 (4)
C9—C8—H8A	119.0	C24—C23—H23A	119.9
O2—C9—C8	122.1 (3)	C22—C23—H23A	119.9
O2—C9—C10	120.9 (3)	C25—C24—C23	118.5 (4)
C8—C9—C10	117.0 (3)	C25—C24—H24A	120.8
C11—C10—C15	119.7 (3)	C23—C24—H24A	120.8
C11—C10—C9	122.6 (3)	C26—C25—F2	119.0 (3)
C15—C10—C9	117.7 (3)	C26—C25—C24	122.9 (4)
C12—C11—C10	118.6 (3)	F2—C25—C24	118.1 (3)
C12—C11—C16	119.4 (3)	C25—C26—C27	118.4 (4)
C10—C11—C16	122.0 (3)	C25—C26—H26A	120.8
C13—C12—C11	121.8 (3)	C27—C26—H26A	120.8
C13—C12—H12A	119.1	C22—C27—C26	120.1 (4)
C11—C12—H12A	119.1	C22—C27—H27A	119.9
C12—C13—C14	119.2 (3)	C26—C27—H27A	119.9
C12—C13—C22	120.9 (3)	O1—C28—H28A	109.5
C14—C13—C22	119.8 (3)	O1—C28—H28B	109.5
C15—C14—C13	119.9 (3)	H28A—C28—H28B	109.5
C15—C14—H14A	120.1	O1—C28—H28C	109.5
C13—C14—H14A	120.1	H28A—C28—H28C	109.5
O1—C15—C14	124.2 (3)	H28B—C28—H28C	109.5
			
C6—C1—C2—C3	0.4 (6)	C13—C14—C15—C10	−1.5 (6)
Br1—C1—C2—C3	179.7 (3)	C11—C10—C15—O1	−179.8 (3)
C1—C2—C3—C4	1.0 (6)	C9—C10—C15—O1	1.8 (5)
C2—C3—C4—C5	−0.4 (6)	C11—C10—C15—C14	1.9 (6)
C3—C4—C5—C6	−1.7 (7)	C9—C10—C15—C14	−176.4 (3)
C2—C1—C6—C5	−2.4 (6)	C12—C11—C16—C21	−47.1 (5)
Br1—C1—C6—C5	178.4 (3)	C10—C11—C16—C21	133.3 (4)
C2—C1—C6—C7	177.6 (4)	C12—C11—C16—C17	133.3 (4)
Br1—C1—C6—C7	−1.7 (5)	C10—C11—C16—C17	−46.2 (5)
C4—C5—C6—C1	3.0 (6)	C21—C16—C17—C18	−2.1 (6)
C4—C5—C6—C7	−176.9 (4)	C11—C16—C17—C18	177.5 (4)
C1—C6—C7—C8	178.2 (4)	C16—C17—C18—C19	−0.4 (6)
C5—C6—C7—C8	−1.9 (7)	C17—C18—C19—F1	−177.1 (3)
C6—C7—C8—C9	178.5 (4)	C17—C18—C19—C20	3.5 (6)
C7—C8—C9—O2	−2.9 (6)	F1—C19—C20—C21	176.6 (3)
C7—C8—C9—C10	176.8 (4)	C18—C19—C20—C21	−4.0 (6)
O2—C9—C10—C11	−55.4 (5)	C19—C20—C21—C16	1.3 (5)
C8—C9—C10—C11	124.8 (4)	C17—C16—C21—C20	1.6 (5)
O2—C9—C10—C15	122.9 (4)	C11—C16—C21—C20	−178.0 (3)
C8—C9—C10—C15	−56.9 (5)	C12—C13—C22—C23	130.6 (4)
C15—C10—C11—C12	−0.6 (5)	C14—C13—C22—C23	−47.2 (5)
C9—C10—C11—C12	177.7 (3)	C12—C13—C22—C27	−47.8 (5)
C15—C10—C11—C16	179.0 (3)	C14—C13—C22—C27	134.4 (4)
C9—C10—C11—C16	−2.7 (6)	C27—C22—C23—C24	0.2 (6)
C10—C11—C12—C13	−1.2 (5)	C13—C22—C23—C24	−178.2 (3)
C16—C11—C12—C13	179.2 (3)	C22—C23—C24—C25	−0.7 (6)
C11—C12—C13—C14	1.7 (5)	C23—C24—C25—C26	0.1 (6)
C11—C12—C13—C22	−176.2 (3)	C23—C24—C25—F2	−179.6 (3)
C12—C13—C14—C15	−0.3 (5)	F2—C25—C26—C27	−179.4 (3)
C22—C13—C14—C15	177.6 (3)	C24—C25—C26—C27	0.8 (6)
C28—O1—C15—C14	−12.1 (5)	C23—C22—C27—C26	0.7 (6)
C28—O1—C15—C10	169.7 (3)	C13—C22—C27—C26	179.1 (3)
C13—C14—C15—O1	−179.6 (3)	C25—C26—C27—C22	−1.2 (6)

### Hydrogen-bond geometry (Å, º)

*Cg*1 and *Cg*2 are the centroids of C1—C6 and C10—C15 rings, respectively.

**Table:**

*D*—H···*A*	*D*—H	H···*A*	*D*···*A*	*D*—H···*A*
C28—H28*A*···F2^i^	0.96	2.51	3.448 (4)	166
C4—H4*A*···*Cg*1^ii^	0.93	2.99	3.712 (5)	136
C20—H20*A*···*Cg*2^iii^	0.93	2.72	3.383 (4)	129
C27—H27*A*···*Cg*1^iv^	0.93	2.95	3.735 (4)	143
C28—H28*B*···*Cg*2^v^	0.96	2.82	3.485 (4)	128

Symmetry codes: (i) *x*+1/2, *y*−1/2, *z*; (ii) *x*, −*y*−1, *z*−1/2; (iii) *x*, *y*+1, *z*; (iv) −*x*−1, −*y*, −*z*; (v) *x*+1/2, *y*+3/2, *z*.
